# Effect of active mind-body movement therapies on older osteoarthritis: a systematic review and meta-analysis of randomized controlled trials

**DOI:** 10.3389/fpubh.2025.1616053

**Published:** 2025-07-08

**Authors:** Congying Pi, Zixi Wang, Lingyu Su, Huaiyu Jian, Junyan Liu, Kun Zhu, Ting Zou, Xiaoyuan Mao, Qinghua Zhang, Zhaoqian Liu

**Affiliations:** ^1^Xiangya School of Pharmaceutical Sciences, Central South University, Changsha, China; ^2^Xiangya Hospital, Central South University, Changsha, Hunan, China

**Keywords:** osteoarthritis, AMBMTs, WOMAC, meta-analysis, SF-36

## Abstract

**Objective:**

To systematically review the literature to quantify and compare the effects of active mind-body movement therapies (AMBMTs) on pain, stiffness, and joint function in older adults with osteoarthritis (OA).

**Methods:**

We searched PubMed, Embase, Cochrane Register of Controlled Trials, Web of Science, ScienceDirect, CINAHL, and PEDro. The outcome measures included the Western Ontario and McMaster Universities Osteoarthritis Index (WOMAC) and the 36-item Short Form Health Survey (SF-36).

**Results:**

A total of 27 studies involving 1781 patients were obtained. The results of meta-analysis showed that compared with the control group, the patients had significantly lower WOMAC pain score (SMD: −0.50, 95%CI: −0.68, −0.32; *p* < 0.01), stiffness score (SMD: −0.71, 95%CI: −1.02, −0.40; *p* < 0.01) and joint function score (SMD: −0.66, 95%CI: −0.85, −0.47; *p* < 0.01).

**Conclusion:**

AMBMTs are a complementary therapy to improve pain in older adult patients with OA, of which Tai Ji is the most effective.

## Highlights

- Osteoarthritis (OA) is a degenerative joint disease with a high incidence among the older adult population.
- Active mind-body movement therapies (AMBMTs) have been recognized and recommended as a new treatment for osteoarthritis.
- Meta-analysis of AMBMTs, showed that this kind of therapy could improve the pain and dysfunction caused by osteoarthritis, thus improving the quality of life of patients.
- Tai Ji can better reduce the pain level of older adult patients.

## 1 Introduction

Osteoarthritis (OA) is the most prevalent joint disease, affecting more than 500 million people worldwide (1). OA is a leading cause of disability in older adults, and this phenomenon becomes worse with the increasing number of aging population (2). OA imparts tremendous humanistic and economic burdens on individuals and society, and with the aging population globally, the clinical and economic burden of OA is increasing further (3). Clinically, the treatment of OA mainly includes non-steroidal anti-inflammatory drug drug therapy, surgical treatment and so on (2). Nevertheless, these approaches merely provide the relief of OA. Meanwhile, several adverse effects occur following pharmacotherapy (4). Exercise therapy is a nonpharmacological modality that reduces pain and improves physical function (5) and is recommended by many expert societies (6).

Different from traditional exercise, mind-body practices are a large and diverse group of procedures or techniques that target brain–body interactions as a way to promote health (7). Mind-body therapy can be classified into three subcategories based on the mode of physical participation, namely static intervention therapies, passive intervention therapies and active mind-body movement therapies (AMBMTs). Static intervention therapies require the body to remain still and relies on cognitive or mental regulation (such as meditation, hypnotherapy, progressive muscle relaxation). In passive intervention therapies, body movement is driven by external forces, and patients do not need the participation of active muscle groups (such as massage therapy, chiropractic manipulation). AMBMTs require patients to independently perform structured movements while integrating conscious concentration and respiratory regulation (such as Tai Ji, Yijinjing, and Yoga) (8, 9). Tai Ji (10), Baduanjin (11), Yijinjing (12), Wuqinxi (13), and Yoga (14) are different types of AMBMTs that can enable people to achieve deep relaxation and peace of mind (15), which can improve the pain and physical function of OA patients.

AMBMT has the characteristics of low intensity and relative safety, which may be a more suitable exercise therapy for older adult patients. At the same time, because the body and mind are integrated in the process of AMBMT, it can relieve the mood in the process of improving the pain of patients. At present, although there are some evidence-based evaluations of AMBMTs therapy for older patients (16, 17), there is no meta-analysis to compare the therapeutic effect of several kinds of AMBMTs on older patients and the effect of AMBMTs on older patients as a whole.

This meta-analysis used data from randomized controlled trials (RCTs) to assess the effects of different psychosomatic AMBMTs on pain, stiffness, physical functioning, mental, and physical health in older patients with OA to provide patients with more optimized exercise treatment program.

## 2 Methods

### 2.1 Study search and selection

This meta-analysis adheres to the Preferred Reporting Items for Systematic Review and Meta-Analysis (PRISMA) guidelines and is registered with PROSPERPO (CRD42024530152), and there was no deviation from the original scheme. We have made a supplementary explanation in the method section. The PRISMA checklist for reporting the meta-analysis results is shown in Supplementary Table 1. We searched PubMed, Embase, Cochrane Register of Controlled Trials, Web of Science, Science Direct, CINAHL, and PEDro for the published articles between their inception and January 2024. In addition, we also screened the references of relevant articles to identify additional published and unpublished records. The search strategy was made for the use of Medical Subject Heading (MESH) terms and correspondence keywords: Tai Ji, Yoga, OA, etc. We also conducted manual searches of grey literature (e.g., conference papers, internal reports, etc.) to avoid missing studies that meet our criteria. Specific search strategies were presented in the Supplementary Table 2.

### 2.2 Inclusion, exclusion criteria, and study outcomes

#### 2.2.1 Inclusion and exclusion criteria

The inclusion criteria of this study were as follows: (1) Patients diagnosed with OA in the lower extremity joints (e.g., knee joint, hip joint, and ankle joint, etc.); (2) Intervention measures in the experimental group were only AMBMTs, including Tai Ji, Qigong, Yoga, Baduanjin, Yijinjing or Wuqinxi; (3) Interventions in the control group included health education, waiting list, self-exercise at home, attention control, and no intervention; and (4) RCTs published in English.

The exclusion criteria were as follows: (1) Intervention measures of the experimental group were AMBMTs combined with other forms of exercise; (2) Intervention measures of the control group were another kind of exercise; (3) Medical records were uneven; (4) Studies without primary outcome data; (5) Cohort studies, case–control studies, and case reports; and (6) Repeatedly published articles, conference abstracts, reviews, and letters.

#### 2.2.2 Outcomes indicators

The primary outcome was Western Ontario and McMaster Universities Osteoarthritis Index (WOMAC) score (18), including pain subscale, physical function subscale, and stiffness subscale. Secondary outcomes were scores on the physical and mental components of the 36-item Short Form Health Survey (SF-36) (19).

### 2.3 Data extraction

Data were extracted and verified independently by two investigators according to inclusion and exclusion criteria. Any differences were resolved through discussion until consensus was reached or a third investigator was consulted. The following data will be extracted for each study: (1) General information: author, year of publication and country of study; (2) Basic information about participants in the intervention and control groups (e.g., age, sex, site of disease and Body Mass Index (BMI)), methods (e.g., randomization, mode of allocation), intervention frequency and duration. (3) Outcomes: WOMAC pain subscale scores, WOMAC physical function subscale scores, WOMAC stiffness subscale scores, SF-36 mental component summary, and SF-36 physical component summary.

### 2.4 Quality assessment

Two authors independently evaluate the methodological quality of the included studies (20) using Cochrane bias risk assessment tool. The details of evaluation included random sequence generation, allocation concealment, blinding of participants and personnel, blinding of outcome assessment, incomplete outcome data, selective reporting, and other bias. We also made a judgment of “low bias risk,” “high bias risk” or “unclear” for each study included.

### 2.5 Statistical analysis

The comparison of outcomes were expressed by standard mean difference (SMD) and 95% confidence intervals (CIs), and the test level was *α* = 0.05. I-squared and Cochran’s Q-statistic test were used to assess the heterogeneity. A fixed-effect model was used, while a random-effects model was used if there was moderate heterogeneity (*I^2^* > 50%, *p* < 0.10). For highly heterogeneous variables, sensitivity analysis or subgroup analysis will be used to explore the source of heterogeneity. Furthermore, we used Egger’s (21) tests to evaluate publication bias, with a visual funnel plot as a complement. Meta-analysis and results were presented using RevMan5.4.1 software (The Cochrane Collaboration, Copenhagen, Denmark) and Stata v12.0 (Stata Corp LP, College Station, TX, United States). A two-sided *p* < 0.05 was considered statistically significant except for the Cochran Q-test. The data used in this study were derived from published literatures, so no additional ethical approval and patient consent were required for this study.

## 3 Results

### 3.1 Search results

**Figure 1:**
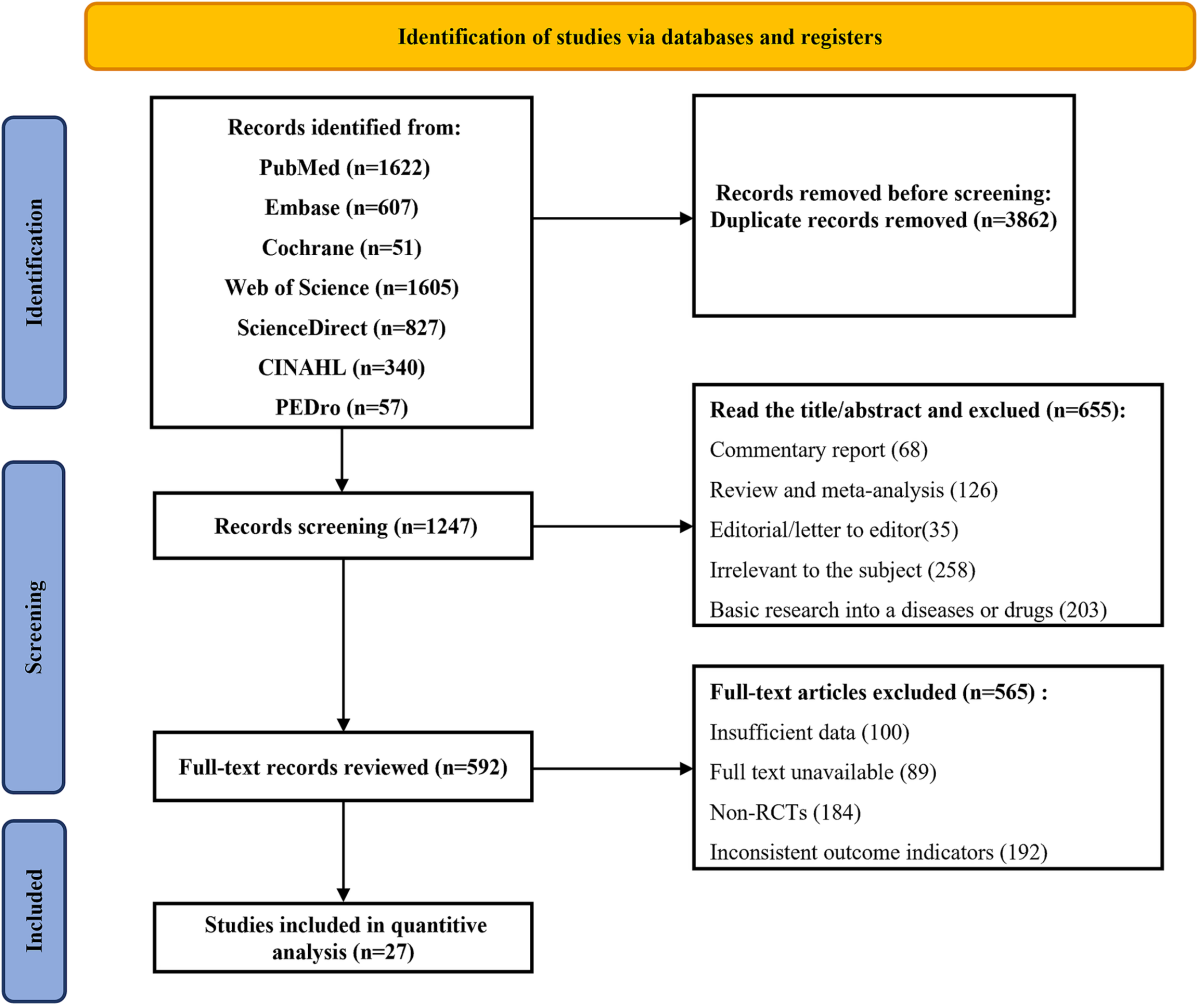
PRISMA flow diagram for search strategy and study selection.

We screened out 5,109 literatures through seven databases. After eliminating duplicate articles, we screened the titles and abstracts of 1,247 literatures, and then excluded 655 literatures. The remaining 592 literatures were scanned according to the inclusion and exclusion criteria. After 565 literatures were excluded, 27 studies were finally included (22–48). They were conducted in five countries, including China (*n* = 11), the United States (*n* = 12), Australia (*n* = 1), India (*n* = 1), and South Korea (*n* = 2) (Figure 1).

### 3.2 Study characteristics

The basic characteristics of the included studies were shown in Supplementary Table 3. There were totally 27 studies (22–48) including 1781 individuals which focused on the therapeutic effects of AMBMTs on OA. Among them, 14 studies (23, 24, 27–31, 35–37, 39, 40, 42, 48) investigated the therapeutic effects of Tai Ji on OA, while 6 studies (25, 26, 32–34, 38) investigated the therapeutic effects of Yoga on OA patients. And the experimental group of 4 studies (22, 41, 45, 46) was treated with Baduanjin exercise therapy. This study (47) reported data on outcomes of Yijinjing. Among the 27 included studies, 24 studies (88.9%) (22–31, 35–48) focused on knee osteoarthritis (KOA), and 3 studies (11.1%) (32–34) included multiple types of joint OA (not subdivided). These studies were published between 2003 and 2022.

### 3.3 Quality assessment

All studies were assessed for risk of bias (Supplementary Figure 4). Twenty-five studies included random sequences as low risk of bias (23–41, 43–48), and two studies did not report random generation (22, 42). For allocation concealment, seven studies did not report information on assignment concealment (22, 25, 28, 35, 37, 42, 45). Four studies lacked blinding of participants and staff (22, 24, 35, 39), and only three studies were unclear (42, 43, 45). For blinding of outcome assessors, one study was at high risk of bias (42), and four studies were unclear (22, 29, 31, 35). Only three studies were at high risk of incomplete data bias (24, 35, 47). Most of the included studies showed a low risk of bias for selective reporting.

### 3.4 Effects of interventions

#### 3.4.1 Effect of AMBMTs on pain

**Figure 2:**
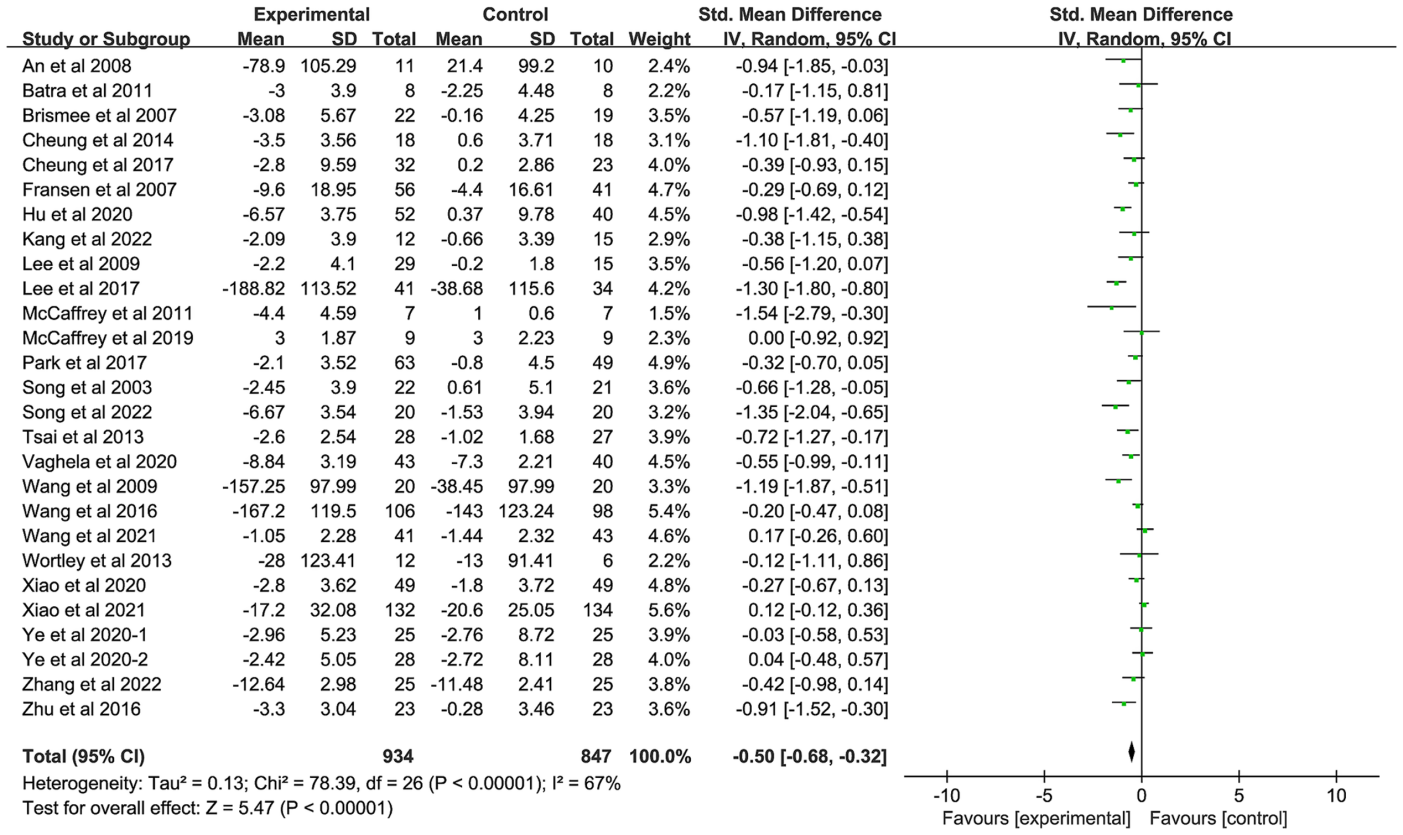
Forest plot showing the effect of AMBMTs on the WOMAC pain.

Twenty-seven studies with a total of 1781 patients were included in this study (22–48). There was moderate heterogeneity among the included studies (*I^2^* = 67%, *p* < 0.01), so a random-effects model was used. The pooled results showed that patients with OA who received AMBMTs had better pain relief than those who did not AMBMTs (SMD: −0.50; 95% CI: −0.68, −0.32; *p* < 0.01) (Figure 2).

#### 3.4.2 Effect of AMBMTs on stiffness

**Figure 3:**
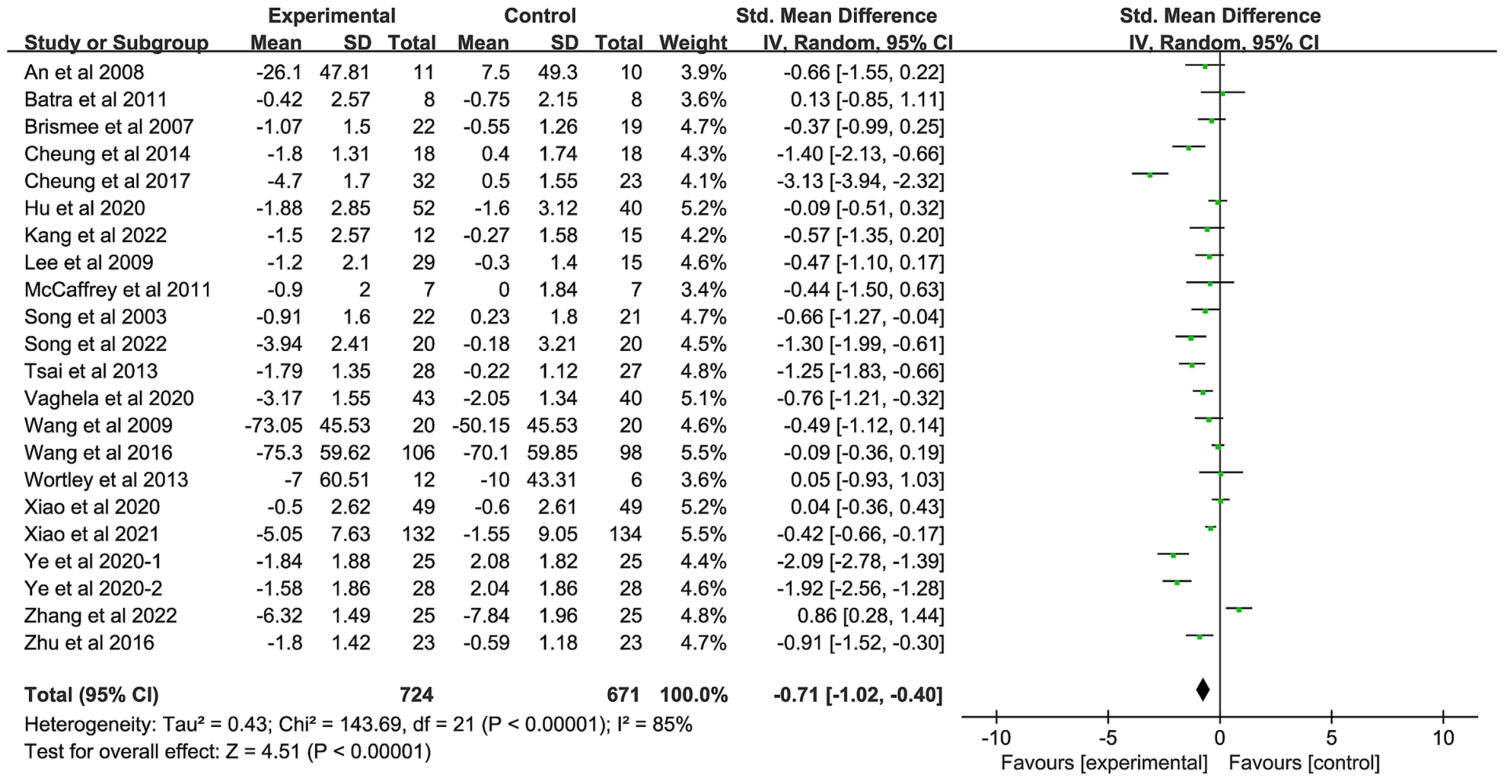
Forest plot showing the effect of AMBMTs on the WOMAC stiffness.

Twenty-two studies with a total of 1,395 patients were included in the study (22–30, 32, 35–40, 42–48). The pooled results showed that patients with OA who received AMBMTs had lower joint stiffness than those who did not receive AMBMTs (SMD: −0.71; 95%CI: −1.02, −0.40; *p* < 0.01) (Figure 3).

#### 3.4.3 Effect of AMBMTs on physical function

**Figure 4:**
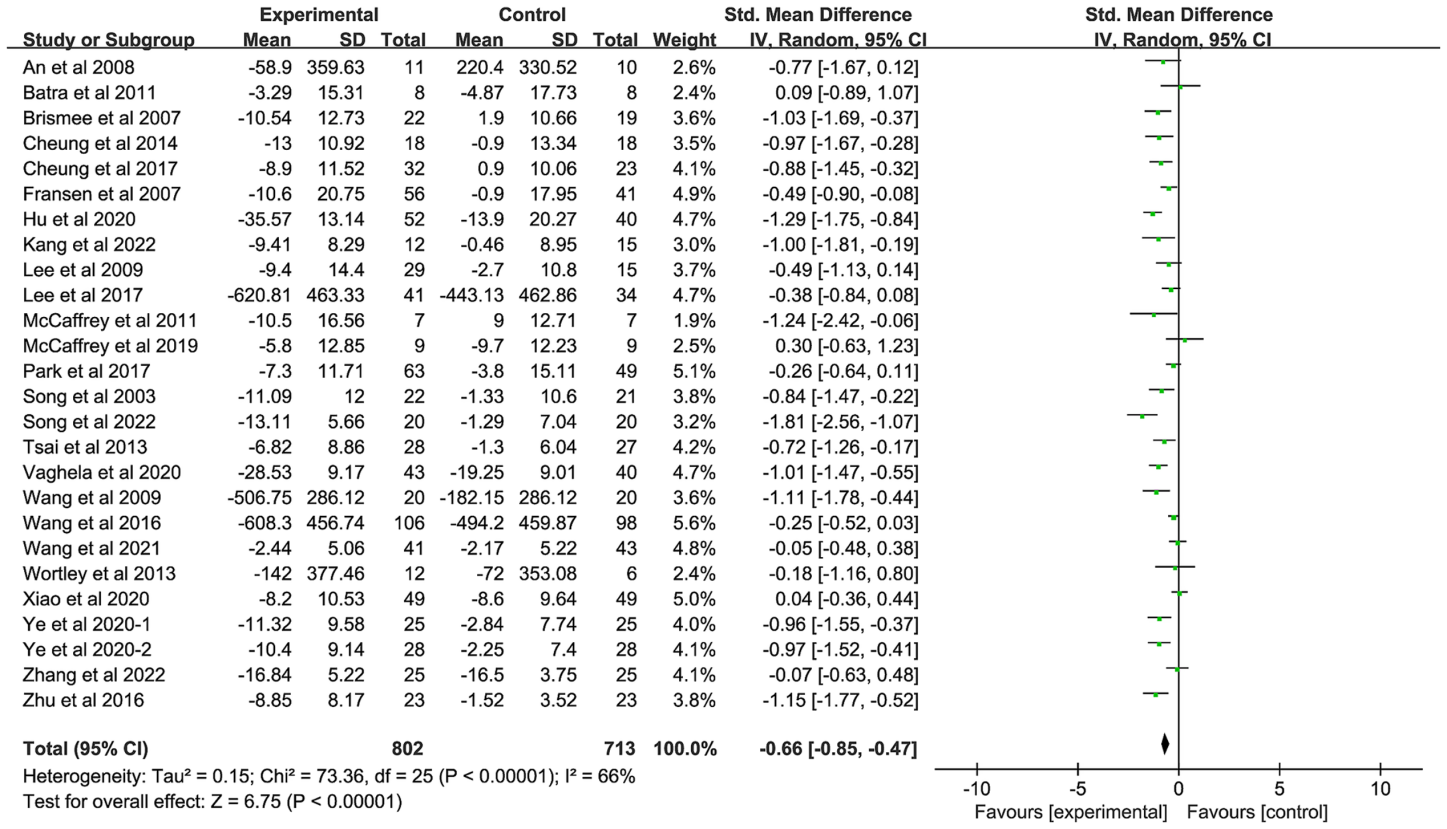
Forest plot showing the effect of AMBMTs on the WOMAC physical function.

Twenty-six studies with a total of 1,515 patients were included in the study (22–43, 45–48). The pooled results showed improved joint function in patients with OA who received AMBMTs compared with patients with OA who did not receive AMBMTs (SMD: -0.66; 95% CI: −0.85, −0.47; *p* < 0.01) (Figure 4).

#### 3.4.4 Effect of AMBMTs on the quality of life

Seven studies assessed the mental component summary (MCS) of the SF-36 scale, and a total of 474 patients were included in the study [22, 30, 31, 36, 39; 40; 47]. The pooled results showed an improvement in mental status in patients with OA who did not receive AMBMTs (SMD: 0.58; 95% CI: 0.22, 0.95; *p* < 0.01) (Supplementary Figure 5).

Physical component summary (PCS) was reported in six studies, and a total of 453 patients were included in the study [30, 31, 36, 39, 40; 47]. The pooled results showed that the mental state of OA patients who did not receive AMBMTs was improved (SMD: 0.62; 95% CI: 0.30, 0.94; *p* < 0.01) (Supplementary Figure 6).

### 3.5 Subgroup analysis results

#### 3.5.1 Subgroup analysis of pain

We first performed a subgroup analysis of movement modalities, which showed a reduction in heterogeneity compared to previous statistics, suggesting that exercise modalities may be one of the sources of heterogeneity in the effect of AMBMTs on pain outcomes in people with OA. At the same time, it was found that among the five exercise methods (Tai Ji, Yoga, Baduanjin, Yijinjing, and Wuqinxi), Tai Ji had a better pain improvement effect (*I^2^*: 60%; SMD: −0.68; 95%CI: −0.92, −0.45; *p* < 0.01, Table 1), followed by yoga (*I^2^*: 35%; SMD: −0.53; 95%CI: −0.84, −0.23; *p* < 0.01, Table 1).

**Table 1:** Results of subgroup analysis affecting WOMAC pain heterogeneity.

Groups	No. of studies	No. of participants	SMD [95%CI]	Heterogeneity		*P*
				*P*	I^2^	
Exercise type						
Tai Ji	14	838	−0.68 [−0.92, −0.45]	<0.01	60%	<0.01
Baduanjin	7	625	−0.08 [−0.30, 0.14]	>0.05	36%	>0.05
Yoga	6	318	−0.53 [−0.84, −0.23]	>0.05	35%	<0.01
Duration time						
≥12 weeks	16	1,098	−0.54 [−0.77, −0.31]	<0.01	70%	<0.01
<12 weeks	11	386	−0.43 [−0.71, −0.14]	<0.01	59%	<0.01
BMI (Mean) (kg/m^2^)						
≥28	7	564	−0.55 [−0.97, −0.12]	<0.01	85%	<0.05
<28	11	495	−0.41 [−0.66, −0.15]	<0.05	55%	<0.01
Age (Mean) (Years)						
≥65	14	853	−0.32 [−0.64, −0.01]	<0.01	74%	<0.05
<65	9	618	−0.62 [−0.93, −0.31]	<0.01	68%	<0.01
Gender						
Females	7	479	−0.71 [−1.23, −0.20]	<0.01	81%	<0.01
Sample size						
≥30	21	1,667	−0.50 [−0.70, −0.31]	<0.01	72%	<0.01
<30	6	114	−0.46 [−0.87, −0.05]	>0.05	13%	<0.05
Frequency						
2 sessions per week	11	688	−0.52 [−0.79, −0.25]	<0.01	59%	<0.01
3 sessions per week	11	617	−0.52 [−0.81, −0.24]	<0.01	66%	<0.01
Control group						
Health education	9	482	−0.71 [−0.96, −0.46]	>0.05	38%	<0.01
Waiting list	7	322	−0.37 [−0.68, −0.06]	>0.05	41%	<0.05
OA position						
KOA	24	1,637	−0.50 [−0.69, −0.31]	<0.01	69%	<0.01

SMD, standard mean difference; BMI, body mass index; OA, osteoarthritis; KOA, knee osteoarthritis.

Second, we performed subgroup analyses for exercise duration, with reduced heterogeneity and better pain relief in exercise duration greater than or equal to 12 weeks (*I^2^*: 70%; SMD − 0.54; 95% CI − 0.77, −0.31; *p* < 0.01, Table 1), suggesting that prolonged exercise relieved pain.

We also analyzed the patient’s own physiology (age and BMI). Despite no evident contribution to the reduction in heterogeneity, it was found that age was younger than 65 years (*I^2^*: 68%; SMD: −0.62; 95% CI: −0.93, −0.31; *p* < 0.01, Table 1).

#### 3.5.2 Subgroup analysis of stiffness

Subgroup analyses were performed as analysis of joint stiffness scores from all included studies found that AMBMTs improved stiffness, but there was a high degree of heterogeneity. Heterogeneity in the Tai Ji group was found to be reduced when subgroup analyses were performed according to exercise patterns, but not compared to the overall improvement (*I^2^*: 60%; SMD: −0.51; 95%CI: −0.78, −0.24; *p* < 0.01, Table 2). However, there was a large heterogeneity in the other exercise mode group, indicating that there may be high heterogeneity in the research literature included in the other exercise mode group.

**Table 2:** Results of subgroup analysis affecting WOMAC stiffness heterogeneity.

Groups	No. of studies	No. of participants	SMD [95%CI]	Heterogeneity		*P*
				*P*	I^2^	
Exercise type						
Tai Ji	12	666	−0.51 [−0.78, −0.24]	<0.01	60%	<0.01
Baduanjin	6	541	−0.67 [−1.41, 0.07]	<0.01	93%	>0.05
Yoga	5	188	−1.44 [−2.53, −0.34]	<0.01	89%	<0.01
Duration time						
≥12 weeks	13	842	−0.65 [−1.06, −0.24]	<0.01	87%	<0.01
<12 weeks	9	553	−0.80 [−1.32, −0.28]	<0.01	84%	<0.01
BMI (Mean ± SD) (kg/m^2^)						
≥28	5	564	−0.43 [−0.79, −0.07]	<0.05	68%	<0.05
<28	10	495	−0.72 [−1.27, 0.17]	<0.01	88%	<0.05
Age (Mean ± SD) (Years)						
≥65	11	735	−0.65 [−1.09, −0.22]	<0.01	85%	<0.01
<65	8	543	−0.67 [−1.21, −0.12]	<0.01	88%	<0.05
Gender						
Females	7	479	−0.79 [−1.10, −0.47]	>0.05	48%	<0.01
Sample size						
≥30	17	1,299	−0.80 [−1.16, −0.45]	<0.01	89%	<0.01
<30	5	96	−0.34 [−0.75, 0.07]	>0.05	0%	>0.05
Frequency						
2 sessions per week	7	386	−0.05 [−0.42, 0.32]	>0.01	56%	>0.05
3 sessions per week	10	533	−0.97 [−1.38, −0.57]	<0.01	79%	<0.01
Control group						
Health education	8	370	−0.99 [−1.61, −0.38]	<0.01	86%	<0.01
Waiting list	6	225	−1.12 [−1.79, −0.45]	<0.01	79%	<0.01
OA position						
KOA	21	1,381	−0.72 [−1.03, −0.40]	<0.01	86%	<0.01

SMD, standard mean difference; BMI, body mass index; OA, osteoarthritis; KOA, knee osteoarthritis.

Subgroup analyses were performed in the same manner as pain outcomes. Heterogeneity was reduced in subgroup analysis by gender, and joint stiffness score was significantly decreased in women after AMBMTs (*I^2^*: 48%; SMD: −0.79; 95%CI: −1.10, −0.47; *p* < 0.01, Table 2), suggesting that patient gender had an impact on outcome heterogeneity. In subgroup analysis, according to the different frequency of exercise per week, twice a week of AMBMTs could not significantly improve joint stiffness (*I^2^*: 56%; SMD: −0.05; 95%CI: −0.42, 0.32; *p* > 0.05, Table 2).

#### 3.5.3 Subgroup analysis of physical function

Finally, we analyzed the joint function outcomes from the above-mentioned subgroup analysis perspectives, suggesting that sample size (less than 30 people) had a significant impact on heterogeneity (Table 3).

**Table 3:** Results of subgroup analysis affecting WOMAC physical function heterogeneity.

Groups	No. of studies	No. of participants	SMD [95%CI]	Heterogeneity		*P*
				*P*	I^2^	
Exercise type						
Tai Ji	14	838	−0.76 [−1.02, −0.50]	<0.01	65%	<0.01
Baduanjin	6	359	−0.76 [−1.02, −0.50]	<0.01	69%	<0.05
Yoga	6	318	−0.67 [−1.08, −0.27]	<0.05	61%	<0.01
Duration time						
≥12 weeks	16	1,098	−0.71 [−0.96, −0.45]	<0.01	74%	<0.01
<12 weeks	10	417	−0.58 [−0.86, −0.29]	>0.05	43%	<0.01
BMI (Mean) (kg/m^2^)						
≥28	6	470	−0.50 [−0.77, −0.24]	>0.05	40%	<0.01
<28	11	579	−0.71 [−1.06, −0.37]	<0.01	74%	<0.01
Age (Mean) (Years)						
≥65	13	997	−0.49 [−0.73, −0.26]	<0.01	64%	<0.01
<65	9	618	−0.78 [−1.12, −0.44]	<0.01	73%	<0.01
Gender						
Females	7	213	−1.09 [−1.39, −0.80]	>0.05	0%	<0.01
Sample size						
≥30	20	1,401	−0.69 [−0.90, −0.48]	<0.01	71%	<0.01
<30	6	114	−0.46 [−0.95, −0.03]	>0.05	37%	0.07
Frequency						
2 sessions per week	11	688	−0.35 [−0.53, −0.17]	>0.05	18%	<0.01
3 sessions per week	10	617	−0.95 [−1.23, −0.68]	<0.01	60%	<0.01
Control group						
Health education	9	482	−0.99 [−1.32, −0.66]	<0.01	62%	<0.01
Waiting list	6	322	−0.70 [−0.93, −0.47]	>0.05	0%	<0.01
OA position						
KOA	22	1,316	−0.68 [−0.89, −0.48]	<0.01	68%	<0.01

SMD, standard mean difference; BMI, body mass index; OA, osteoarthritis; KOA, knee osteoarthritis.

### 3.6 Sensitivity analysis and risk of bias assessment

As shown in Supplementary Figure 7, we eliminated the main outcome indicators one by one for sensitivity analysis, and found that there was no statistical difference between the results before and after elimination, which indicated that the results of all included studies were more reliable. Secondly, we used a funnel chart to evaluate publication bias and Egger’ test to detect the symmetry of funnel chart. From the funnel chart results, there is asymmetry in the funnel chart of pain and physical function (Supplementary Figure 8). According to Egger’s test, the pain score of WOMAC is t = −1.97 (95%CI: −4.13, −0.88, *p* < 0.01, Supplementary Figure 9), stiffness result is t = −0.52 (95% CI: −1.97, 0.15, *p* > 0.05, Supplementary Figure 9), and physical function is t = −2.11 (95%CI: −4.08, −0.49, *p* < 0.05, Supplementary Figure 9). Egger’ test is consistent with the funnel chart results, which shows that there is publication bias in the study of pain and physical function.

## 4 Discussion

OA is one of the chronic diseases that plague the older adult, which greatly affects the life quality of patients. The treatment of OA has always been on the agenda, and in addition to drug treatment, exercise therapy has also received extensive attention in recent years (49). Among them, Tai Ji, Yoga, Baduanjin and other slow and accurate action training can enhance proprioception and muscle coordination, thus improving physical function. Secondly, it can improve mood and enhance pain tolerance by adjusting the autonomic nervous system (such as increasing vagus nerve tension) and reducing stress response (such as reducing cortisol level), which can be applied to different people (50–53). Although there is evidence supporting the efficacy of AMBMTs on older adult patients, these studies mainly focus on the efficacy of a single exercise mode, and little attention is paid to the efficacy of this type of AMBMTs. Therefore, our current work provides an important supplement in the aspect of the published meta-analyses by pooling the published RCTs in the relevant area.

Results from a recent meta-analysis (16) showed that mind-body exercises (Tai Ji, Baduanjin, and Yoga) significantly improved pain (SMD: -0.65; 95% CI: −0.87, −0.42), stiffness (SMD: −0.75; 95%CI: −1.05, −0.45). The article explores more types of exercise, including not only the traditional mind-body movement in China but also the AMBMTs of yoga, which originated in India. Among them, for traditional Chinese sports, in addition to the inclusion of Tai Ji, which has attracted much attention, it also pays attention to the three sports of Baduanjin, Yijinjing, and Wuqinxi, which are slightly less well-known but have their own characteristics. Another meta-analysis, which also used WOMAC as the sole measure (10), showed that Tai Ji was associated with better pain relief (SMD: −0.51; 95%CI: −0.89, −0.13; *p* < 0.01), and Baduanjin had an improvement in joint stiffness (SMD: −1.30; 95%CI: −2.32, −0.28; *p* < 0.05) and improved somatic function (SMD: −0.52; 95%CI: −0.97, −0.07; *p* < 0.05). But from the results of subgroup analysis of exercise types in this study, Tai Ji not only had a good effect on pain relief (SMD: −0.68; 95%CI: −0.92, −0.45; *p* < 0.01), but also improved physical function (SMD: −0.76; 95%CI: −1.02, −0.50; *p* < 0.01), whereas Yoga had a better advantage in improving joint flexibility (SMD: −1.44; 95% CI: −2.53, −0.34; *p* = 0.01). The reason for the discrepancy in the results may be that this study was limited to patients with OA of lower extremity joints, while published studies have focused on the population of patients with knee arthritis. Considering the moderate heterogeneity shown in this study, in addition to conducting subgroup analyses for different exercise types of AMBMTs, we also studied other factors, such as intervention time, patient age, sample size, etc. Among them, motor typing can well explain the heterogeneity existing in the study. Meanwhile, subgroup analysis based on sample size can significantly reduce heterogeneity. This might be because the wide confidence intervals of small-sample studies mask group differences, while the precise data of large samples bring subgroup differences (such as patient age and BMI) to the surface. This results suggest that AMBMTs in patients older than 65 years still has some improvement effect, especially in improving joint stiffness, which is similar to that of patients younger than 65 years (SMD: −0.65; 95% CI: −1.09, −0.22; *p* < 0.01), probably due to the fact that this type of low-intensity, low-load traditional cardio exercise requires the whole body to be in a relaxed state, which can help improve cardiopulmonary function and physical agility in older adult patients. Therefore, it is recommended that older adult patients try and practice this kind of exercise appropriately.

When patients with OA suffer from chronic pain, their mental state may be affected, further impacting their lives. Therefore, we selected the Physical Health (PCS) and Mental Health (MCS) scores of SF-36 to evaluate the impact of AMBMTs therapy on the quality of life of older adult patients with OA. The results showed that the SF-36 scores of OA older adult patients were significantly improved, which was consistent with the results of a previous study (6). However, due to the lack of this indicator in yoga-related studies, it mainly suggests that traditional Chinese mind-body exercises (Tai Ji, Baduanjin, and Yijinjing) can be able to improve the quality of life of OA older adult patients.

The following limitations of this study should be noted. First, the inclusion of individual RCTs had issues of unclear allocation methods and reporting bias, which reduced confidence in the overall results. Second, due to the limitation of the quality of the original data reports, subgroup analyses based on baseline OA severity (KL classification or WOMAC score) were not conducted for all included studies in this study. The combined effect size may represent the “average value” of subgroups with different severity levels, masking the potential heterogeneity of therapeutic effects (such as exercise being more effective for early OA but less effective for late OA). Last, because 24 weeks was the maximum duration of treatment in the included studies, our results can only be used as a basis for the short-term treatment of OA with AMBMTs, but the long-term benefits of this type of exercise are unclear. So future RCTs and meta-analyses must systematically collect and report the baseline severity of OA in patients (standard indicators such as KL classification and WOMAC), and incorporate severity subgroup analyses as a key part of preset analyses or sensitivity analyses to provide more accurate and personalized evidence. In addition, long-term follow-up of ≥ 1 year is needed to evaluate the sustained benefit and dose–response relationship of AMBMTs, and to design a step-by-step intervention scheme (such as short-term reinforcement and long-term maintenance) to balance the immediate effect and sustainability, so as to provide better strategies for clinical practice. Furthermore, various geographical and cultural backgrounds may lead to different understanding angles and different understanding of the same type of sports. A large class of sports will produce different factions, and the specific sports postures in different factions are also different. For example, there are differences between different schools in action intensity and breathing rhythm, which may lead to the heterogeneity of exercise intervention effect. For example, the high-intensity burst action of Chen Tai Ji may improve muscle strength more significantly, while the uniform style of Yang Tai Ji may be more conducive to cardiovascular regulation, so even if the same type of exercise is used, the therapeutic effect may be different due to different schools (54). Therefore, it is difficult to come up with an individualized treatment plan for different groups of people, involving specific sports genres, sports frequencies and sports duration.

## 5 Conclusion

AMBMTs (especially Tai Ji) can improve pain and dysfunction caused by OA, thus improving patients’ quality of life. However, due to the methodological limitations of some of the included studies, more high-quality RCTs with large samples, multicenter, and conducted over a long period of time are needed for further validation and support.

## Data Availability

The raw data supporting the conclusions of this article will be made available by the authors, without undue reservation.
